# A Monte Carlo algorithm to improve the measurement efficiency of low-field nuclear magnetic resonance

**DOI:** 10.1038/s41598-023-37731-8

**Published:** 2023-06-29

**Authors:** Pan Guo, Ruoshuang Zhang, Jiawen Zhang, Junhao Shi, Bing Li

**Affiliations:** 1 College of Physics and Electronic Engineering, Chongqing Normal University, Chongqing, China; 2 Urumqi Power Supply Company, State Grid Xinjiang Electric Power Co, LTD, Urumqi, China

**Keywords:** Energy science and technology, Engineering, Physics

## Abstract

Nuclear magnetic resonance (NMR) has shown good applications in engineering fields such as well logging and rubber material ageing assessment. However, due to the low magnetic field strength of NMR sensors and the complex working conditions of engineering sites, the signal-to-noise ratio (SNR) of NMR signals is low, and it is usually necessary to increase the number of repeated measurements to improve the SNR, which means a longer measurement time. Therefore, it is especially important to set the measurement parameters appropriately for onsite NMR. In this paper, we propose a stochastic simulation using Monte Carlo methods to predict the measurement curves of ${\mathrm{T}}_{1}$ and ${\mathrm{D}}_{0}$ and correct the measurement parameters of the next step according to the previous measurement results. The method can update the measurement parameters in real time and perform automatic measurements. At the same time, this method greatly reduces the measurement time. The experimental results show that the method is suitable for the measurement of the self-diffusion coefficient D_0_ and longitudinal relaxation time T_1_, which are frequently used in NMR measurements.

## Introduction

Nuclear magnetic resonance (NMR) can be used to explore the structure and properties of substances from the microscopic level through non-destructive methods and is now widely used in the fields of medicine, chemistry, materials, biology, petroleum, geology^1^, etc. The application of portable NMR sensors and unilateral magnetic resonance devices has attracted the attention of many researchers^2–5^. However, the low magnetic field strength and uniformity and complex working conditions result in a low signal-to-noise ratio (SNR), long measurement time and low measurement efficiency. These are currently urgent problems that need to be solved. A more effective way is to optimize the measurement process and set the measurement parameters appropriately to improve the efficiency.

There are some reports on the parameter optimization algorithm in NMR measurements. Some scholars have noticed the influence of the magnetic resonance measurement parameters on the measurement results, but their research mainly focuses on the frequency domain^6–9^, that is, exploring the data acquisition method that can reduce the measurement time and ensure that the frequency domain inversion spectrum has sufficient precision^10–14^. In 2017, Xing D. et al.^15^ presented an adaptive method for determining an acquisition parameter *t*_*0*_ in a modified CPMG sequence for measuring the internal magnetic field gradient distribution of samples. This method can reduce the difficulties of operating T_2_-G experiments. In 2018, A. Reci^16^ proposed an optimization method for the measurement parameters of the NMR liquid self-diffusion coefficient based on the Cramér-Rao lower bound (CRLB) theory, which assumes that the self-diffusion coefficient of the sample satisfies the log-normal distribution. In 2019, A. Reci ^17^ further improved the sampling method based on CRLB theory, making it also applicable to signal acquisition with double exponential decay. In 2021, Guest et al. studied the relationship between the experimental parameters and SNR for diffusion coefficient measurement^18^.

In recent years, NMR has been widely used in industrial automation^19,20^, but few studies have provided a more general time-domain sampling strategy for T_1_ and D_0_ measurement experiments in on-site NMR. At the same time, some users of NMR instruments may not have background knowledge of the NMR principles, so it is difficult for them to adjust the measurement parameters. Therefore, automatic and intelligent measurement algorithms are wanted by users. In this paper, we introduce a Monte Carlo algorithm-based intelligent search method for NMR measurement parameters. This method can automatically set the key parameters in the T_1_ and D_0_ measurements. The experimental results demonstrate that the method could achieve a 3–4 times acceleration effect in the T_1_ and D_0_ measurement experiments, and the systematic error between the results and the accurate value was less than 5%.

## Algorithm introduction

### Introduction to the Monte Carlo method

The Monte Carlo method, also known as the statistical simulation method, is a numerical calculation method guided by probabilistic statistical theory^21–29^. The characteristic of this method is the random sampling of the measurement variable, and an approximate result is calculated in real time for the NMR measurement constrained with the available dataset. In this paper, an intelligent search method for NMR measurement parameters based on the Monte Carlo algorithm is proposed to achieve the measurements of T_1_ and D_0_.

### Measurement sequence

**Figure 1:**
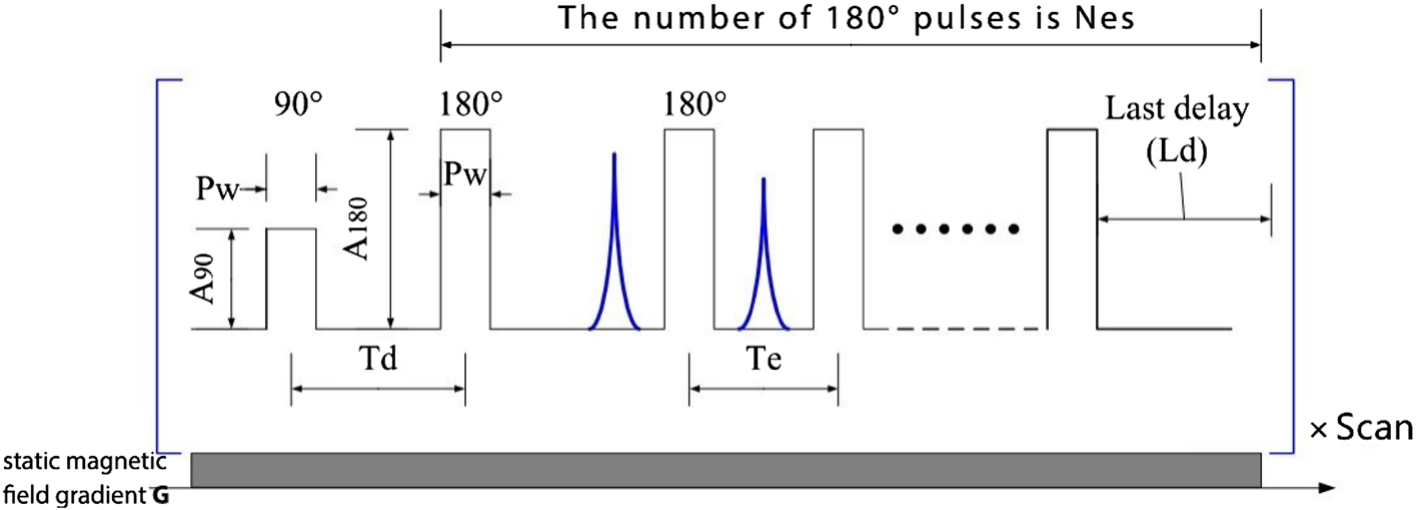
SESG-CPMG measurement of D_0_ sequences and their parameters.

Figure 1 shows the static gradient spin echo (SGSE) sequence 1 for D_0_ measurement in low-field NMR when a static gradient magnetic field is present^30^. where the time interval between the 90° and 180° pulses (${\mathrm{T}}_{\mathrm{d}}$) is variable. When measuring the diffusion properties, ${\mathrm{T}}_{\mathrm{d}}$ is increased at each ${\mathrm{T}}_{\mathrm{d}}$ point, and the spin echoes are recorded. Therefore, the diffusion decay curve of a liquid molecule under a gradient magnetic field can be fitted. To improve the SNR, the sequence shown in Fig. 1 needs to be repeated several times and superimposed, and it is therefore quite time-consuming.

Currently, the common way to increase the ${\mathrm{T}}_{\mathrm{d}}$ value is uniform stepping, if N signals need to be measured to fit the curve of D_0_. ${\mathrm{T}}_{\mathrm{d}}$ is set at a uniformly increasing step rate.

The diffusion curve is estimated by using these measured values, which consumes considerable time. At the same time, some users of NMR instruments may not have background knowledge of the NMR principles, so it is difficult for them to adjust the measurement parameters. Therefore, automatic and intelligent measurement algorithms are wanted by users.

**Figure 2:**
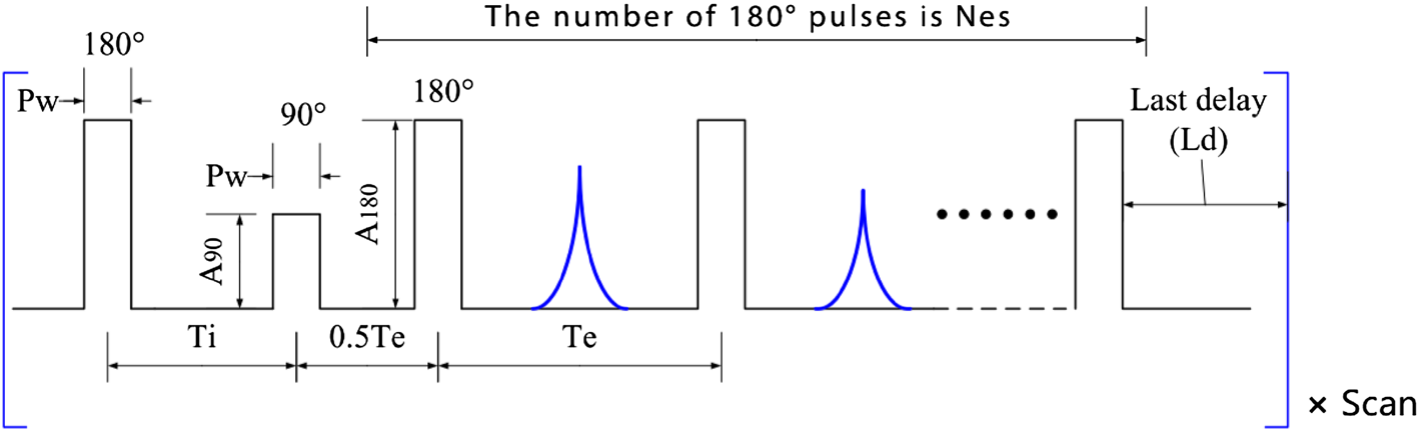
IR-CPMG measurement of T_1_ sequences and their parameters.

The inversion recovery (IR) sequence used to measure T_1_ is shown in Fig. 2. Similar to the SGSE sequence and variable T_d_, ${T}_{i}$ is varied to measure the T_1_ recovery of the sample.

### Method

In ${\text{T}}_{1}$ and ${\text{D}}_{0}$ measurements, adjusting ${\text{T}}_{\text{d}}$ and ${\mathrm{T}}_{\mathrm{i}}$ during the measurement needs to be performed. In our method, many diffusion curves and ${\text{T}}_{1}$ decay curves are randomly generated by Monte Carlo simulations, and then the unreasonable curves are excluded according to the data already measured, and the remaining curves can be used to estimate the measurement parameters for the next step. The detailed steps of the algorithm are as follows.

First, the initial range of B and ${\text{D}}_{0}$ are determined in Eq. (1), and the two-dimensional space determined by the initial ranges of these two parameters is called the initial parameter space. The initial parameter space is set to B ∈ [0, 2], ${D}_{0}$∈[10^–6^ mm^2^/s, 10^–2^ mm^2^/s]. The ${D}_{0}$ range can cover most of the self-diffusion coefficients of the tested samples. The variation range is [2 ms, 180 ms], which can reflect the attenuation curve of all samples in the above ${\text{D}}_{0}$ range. A Monte Carlo sampling method is used to randomly select a specified number of data points in this initial parameter space and thus draw a cluster of t-$\mathrm{f}(\mathrm{t})$ curves to calculate the uncertainty of the model at this point (expressed as the product of the variance of B and D0 in this space). f(t) is the diffusion decay curve, and t is the abscissa of the measured data points. The most divergent time point ${\mathrm{t}}_{1}$ in this curve cluster is selected as the first sampling point, and the actual corresponding measurement value $\mathrm{f}{(\mathrm{t}}_{1})$ is obtained by measurement. From this measurement dataset, (${\mathrm{t}}_{1}$, $\mathrm{f}{(\mathrm{t}}_{1})$) constitutes the first data point of the dataset, and the initial parameter space of B and D_0_ is constrained under the constraints of this data point (i.e. only the parameter space that matches the (${\mathrm{t}}_{1}$, $\mathrm{f}{(\mathrm{t}}_{1})$) data point is retained), which in turn yields the B and D_0_ parameter subspaces. Similarly, the t-$\mathrm{f}(\mathrm{t})$ curve cluster is plotted in this parameter subspace, and the model uncertainty is calculated, while the point ${\mathrm{t}}_{2}$, which makes the curve cluster most divergent, is still selected as the next sampled data point, and the measurement is performed. At this time, the data space is expanded to ((${\mathrm{t}}_{1}$, f(${\mathrm{t}}_{1}$), (${\mathrm{t}}_{2}$, f(${\mathrm{t}}_{2}$))), and the parameter space of B and D_0_ will be further reduced under the constraint of these two measurement data points. The above steps are repeated until the model uncertainty no longer significantly decreases; then, the parameter space of B and D_0_ under the data space constraint consisting of all measured data will be close to the true B and D_0_ values.

The diffusion decay curve $\mathrm{f}\left(\mathrm{t}\right)$ satisfies the following form^30^:

1 $$\begin{array}{c}f\left(\mathrm{t}\right)=B*{\mathrm{e}}^{-{\left(\frac{\mathrm{t}}{{\mathrm{D}}_{0}}\right)}^{3}},\end{array}$$

This equation is correct for the constant gradient case, where the parameter vector A =$\left[{\mathrm{D}}_{0},\mathrm{B}\right]$ is to be determined. t is the abscissa of the measured data points. Considering the form of Eq. (1), if there is no measurement error, $\mathrm{B}$ should be equal to the peak of the measured echo, and the maximum $\mathrm{Max}\left({\dot{\mathrm{y}}}_{\mathrm{i}}\right)$, ${\dot{\mathrm{y}}}_{\mathrm{i}}$ is the real measurement data. Considering the measurement error, the parameter search range can be set to $\left[\mathrm{Max}\left({\dot{\mathrm{y}}}_{\mathrm{i}}\right)\pm 5\upsigma \right]$; and the ${\mathrm{D}}_{0}$ of common samples is between 0 and ${10}^{-8}{\mathrm{cm}}^{2}\cdot {\mathrm{s}}^{-1}$. Therefore, ${\mathrm{D}}_{0}$ and B are set to obey the uniform distribution in their respective intervals:

2 $$\begin{array}{c}\left\{\begin{array}{c}{\mathrm{D}}_{0}\sim U\left[0,{10}^{-8}\right]\\ B\sim U\left[\mathrm{Max}\left({\dot{\mathrm{y}}}_{\mathrm{i}}\right)-5\upsigma ,\mathrm{Max}\left({\dot{\mathrm{y}}}_{\mathrm{i}}\right)+5\upsigma \right]\end{array}\right.,\end{array}$$

Multiple random sampling according to the above distribution is performed.

3 $$\begin{array}{c}{{\sum }_{\mathrm{i}=1}^{\mathrm{N}}\left[{\dot{\mathrm{y}}}_{\mathrm{i}}-\mathrm{B}*{\mathrm{e}}^{-{\left(\frac{\mathrm{t}}{{\mathrm{D}}_{0}}\right)}^{3}}\right]}^{2}<N{\upsigma }^{2},\end{array}$$

When there are sufficient curves that satisfy the condition of Eq. (3), ${\mathrm{DY}}_{\mathrm{t}}$ can be calculated, and then, it can be substituted into ${\dot{\mathrm{t}}}_{\mathrm{i}+1}=\mathrm{t}|{\mathrm{DY}}_{\mathrm{t}}=\mathrm{Max}\left({\mathrm{DY}}_{\mathrm{t}}\right)$ to obtain ${\dot{\mathrm{t}}}_{\mathrm{i}+1}$. ${\mathrm{T}}_{\mathrm{d}}$ is set to ${\mathrm{t}}_{\mathrm{i}+1}$ to obtain the new measured value of ${\dot{\mathrm{t}}}_{\mathrm{i}+1}$, and it is fit by least squares to obtain ${\widehat{\mathrm{a}}}_{\mathrm{i}+1}$. The iterations are stopped when $|{\widehat{\mathrm{a}}}_{0\left(\mathrm{i}\right)}-{\widehat{\mathrm{a}}}_{0\left(\mathrm{i}-1\right)}|<\upvarepsilon$ is satisfied. The measurement of ${\mathrm{T}}_{1}$ was performed similarly.

**Figure 3:**
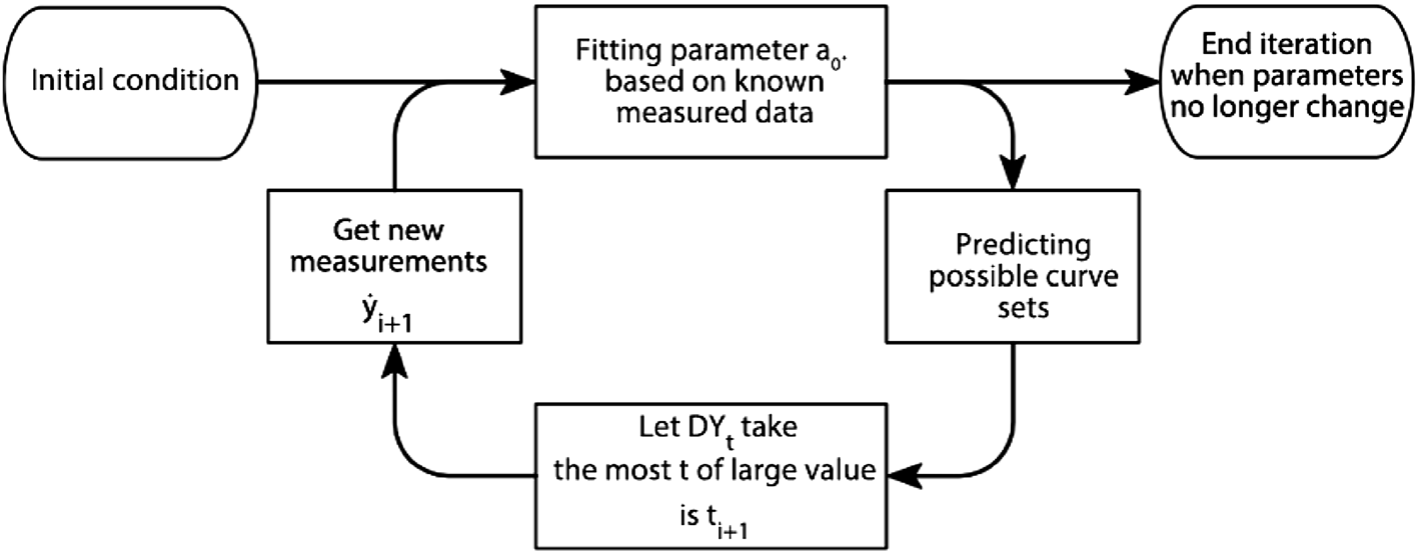
Algorithm flow chart.

The entire algorithm flow is shown in Fig. 3.

## Experimental verifications

**Figure 4:**
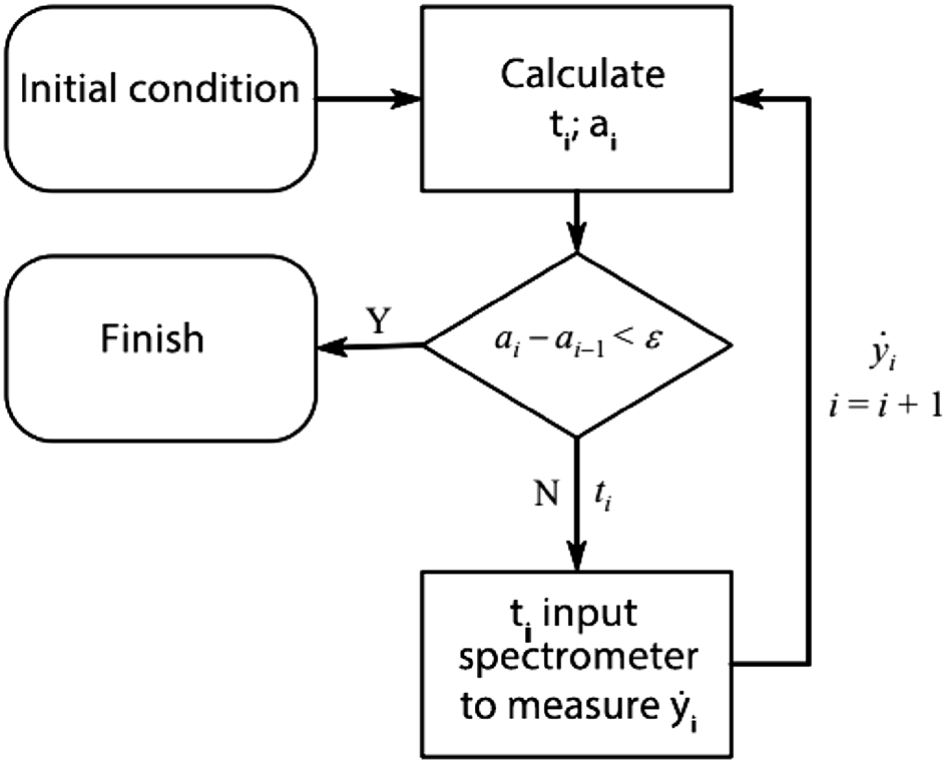
Measurement flow chart.

The equipment used in the experiment was a spectrometer (KEA2, Magritek Inc. New Zealand), RF power amplifier (BT00500 ALPHA-SA, Tomoco Inc., Australia) and unilateral nuclear magnetic resonance sensor (130 mT). To verify the effectiveness of the above algorithm, the diffusion coefficient of pure water was measured at points taken with the algorithms ${\mathrm{D}}_{0}$ and ${\mathrm{T}}_{1}$. Moreover, ${\mathrm{t}}_{\mathrm{i}}$ obtained by the algorithm is inputted into the programmable spectrometer, ${\dot{\mathrm{y}}}_{\mathrm{i}}$ is obtained from the actual measurement, and then, ${\dot{\mathrm{y}}}_{\mathrm{i}}$ is returned to the algorithm to calculate ${\mathrm{t}}_{\mathrm{i}+1}$. This process is shown in Fig. 4.

**Figure 5:**
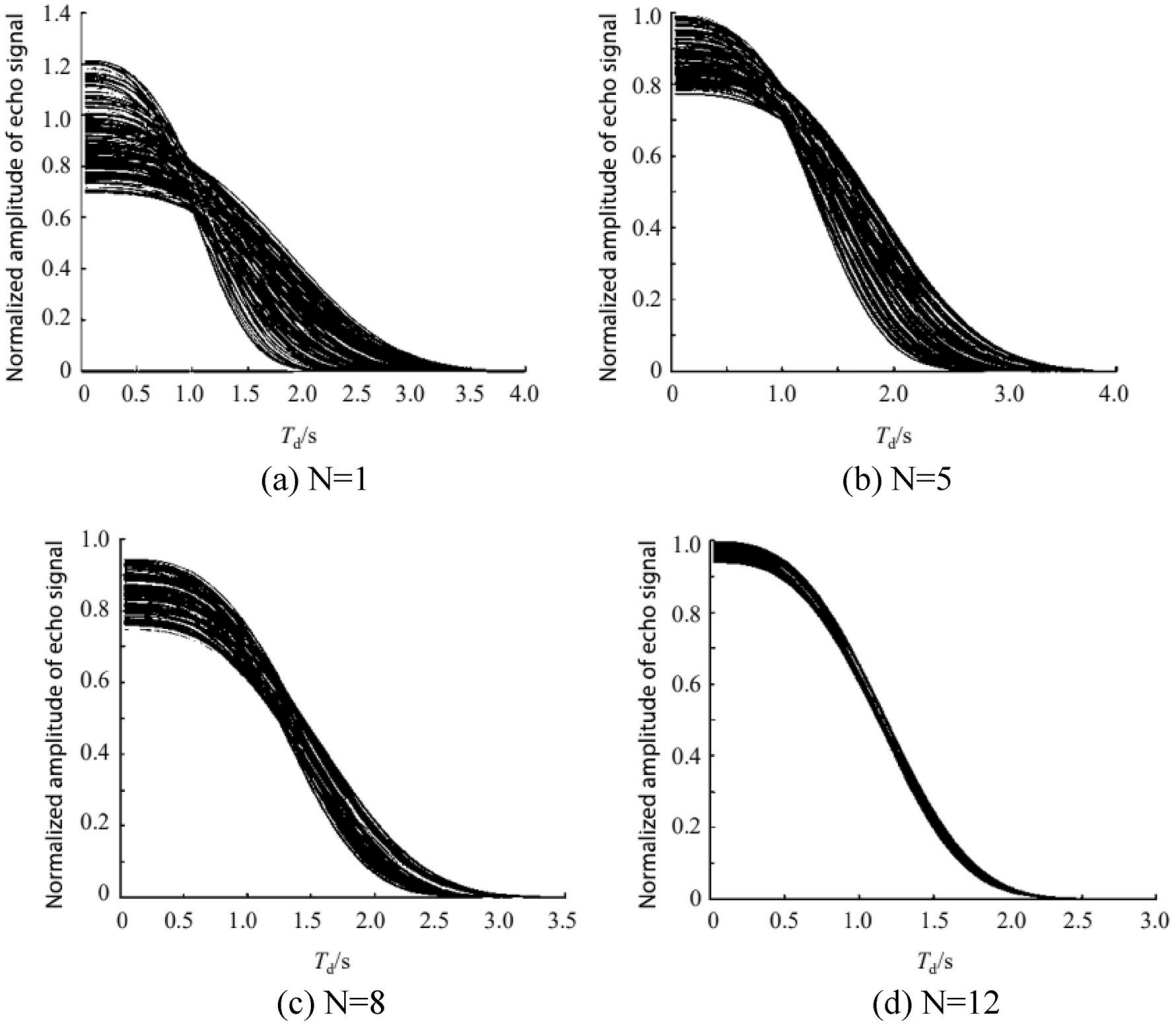
${\mathrm{D}}_{0}$ Convergence of the predicted dataset in the measurement with increasing measurement data.

**Figure 6:**
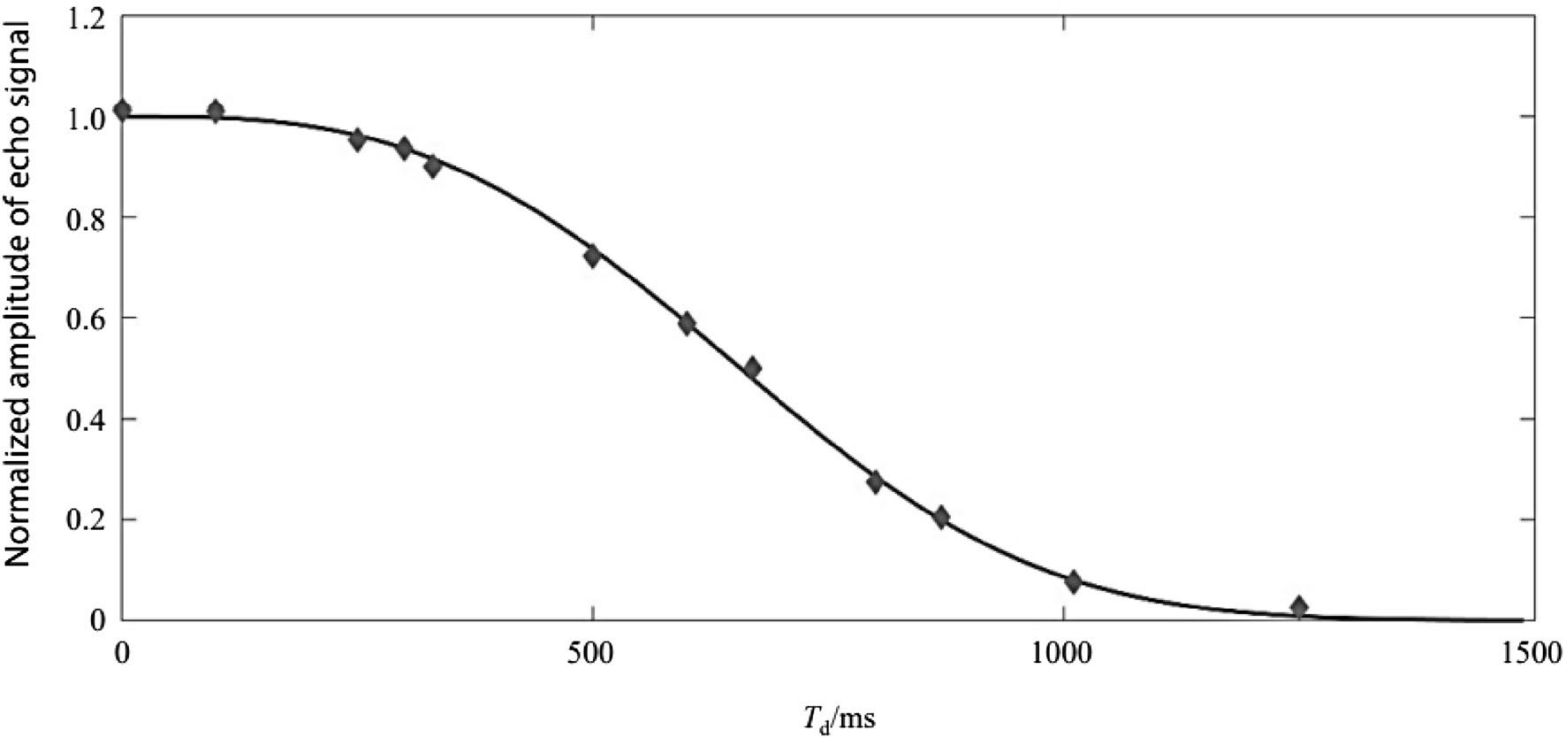
Pure water measured diffusion curve.

The upper limit of error $\upvarepsilon ={10}^{-10}$ is set, and the algorithm stops iterating after 12 points are measured. With the increase in measurement data, the prediction dataset continues decreasing, giving the prediction dataset when the known data are 1, 5, 8, and 12, respectively, as shown in Fig. 5, and the diffusion curve plotted by the actual measurement data as shown in Fig. 6.

**Figure 7:**
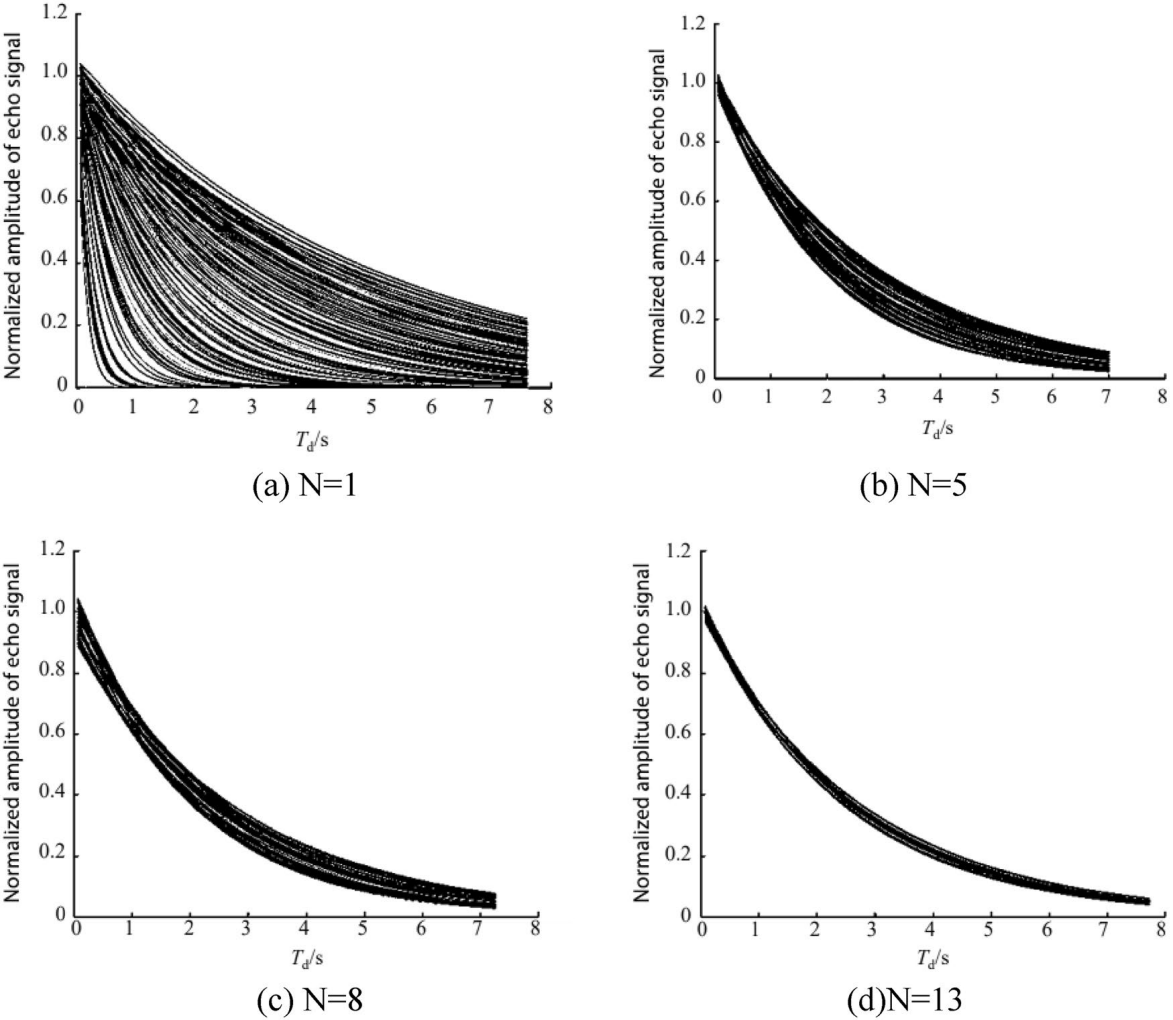
Convergence of the prediction dataset with an increasing measurement data in the T_1_ measurement.

**Figure 8:**
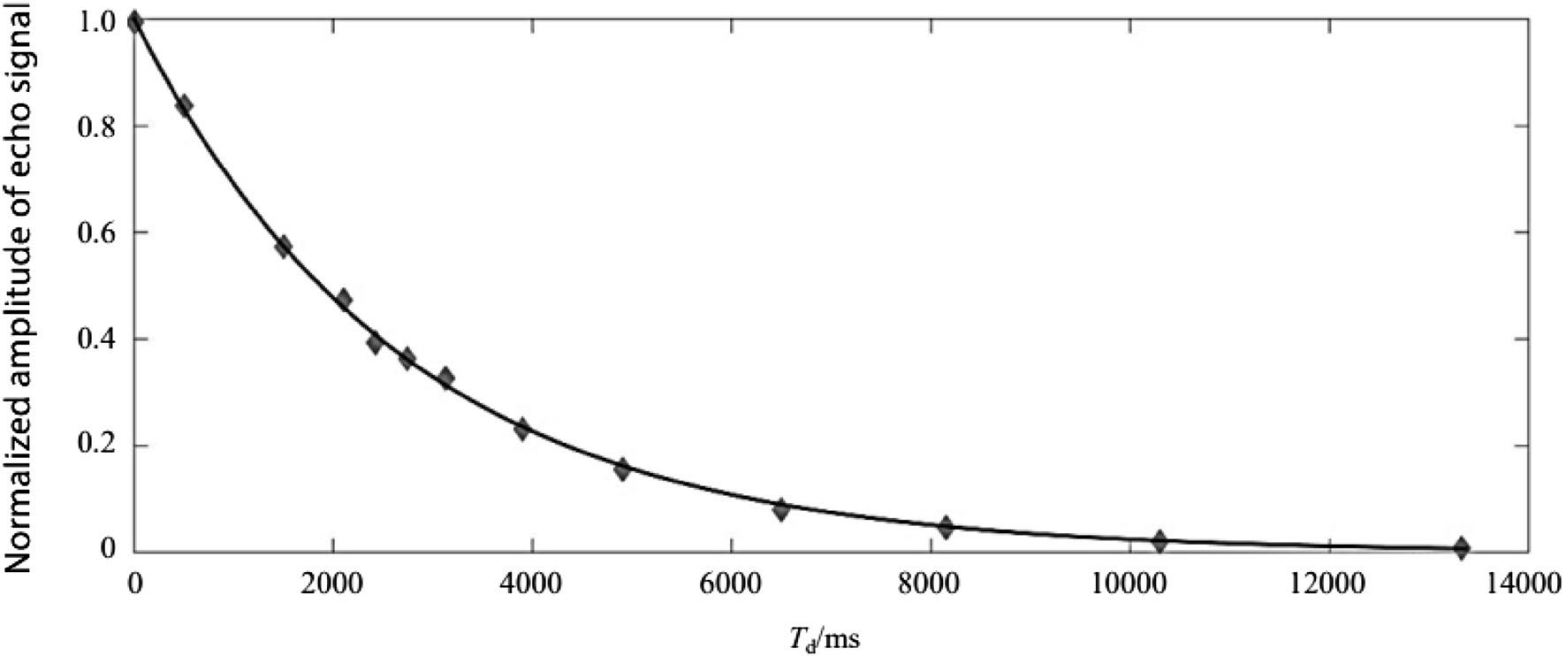
Pure water actual measurement ${\mathrm{T}}_{1}$.

The same algorithm is used to measure ${\mathrm{T}}_{1}$ of the pure water, the upper error limit is set to $\upvarepsilon ={10}^{-10}$, and the algorithm stops iterating after measuring 13 points. The predicted dataset with the known number of Data Points 1, 5, 8, and 13 is shown in Fig. 7, and the actual measured ${\mathrm{T}}_{1}$ curve is shown in Fig. 8.

Table 1 shows the data of D_0_ and T_1_ obtained by the Monte Carlo intelligent algorithm and manual measurements using the same experimental equipment with pure water and glycerol as samples at 20 °C room temperature, respectively.

**Table 1:** D_0_ and T_1_ of deionized water and glycerine by the Monte Carlo method and manual, where M-C replaced Monte Carlo, and points represents the measurement points in the D_0_ and T_1_ experiments.

	D_0_ (mm^2^/s)		Points		T_1_ (ms)		Points	
	M-C	Manual	M-C	Manual	M-C	Manual	M-C	Manual
Water	2.49 × 10^–3^	2.35 × 10^–3^	4	15	2653	2650	4	15
Glycerine	0.124 × 10^–3^	0.12 × 10^–3^	3	15	227	230	3	15

To evaluate the accuracy and stability of the Monte Carlo optimization-based algorithm in the D_0_ and ${\mathrm{T}}_{1}$ measurement experiments, 10 repeated D_0_ and ${\mathrm{T}}_{1}$ measurements were performed on pure water and glycerol samples, respectively. The deviation of the mean value of the 10 replicate measurements relative to the manual measurements was used to measure the systematic error of the algorithm, and the standard deviation of the 10 replicate measurements was used to measure the accuracy of the algorithm^30^, as shown in Table 2.

**Table 2:** Bias and standard deviation of D_0_ and T_1_ by repeating the measurement 10 times.

	Bias		Standard deviation	
	D_0_	T_1_	D_0_	T_1_
Water	− 0.34%	− 0.48%	1.75%	2.68%
Glycerine	− 0.83%	0.96%	1.66%	2.23%

The Monte Carlo-based optimization algorithm was experimentally demonstrated to be well adapted to two D_0_, pure water and glycerol, in ${\mathrm{T}}_{1}$ samples with values tens of times different, and the systematic error relative to the manually measured values is less than 1%. Considering the effect of temperature, it can be considered that the ${\mathrm{D}}_{0}$ and ${\mathrm{T}}_{1}$ parameters of the measured pure water under this algorithm are basically accurate.

In addition, with the algorithm optimization, the D_0_ and ${\mathrm{T}}_{1}$ measurement experiments can be completed with only 3 to 4 data points and achieves a speed increase of approximately 6 times compared to manual measurements of 15 to 25 data points, even without taking into account the manual process of repeatedly adjusting the measurement parameters, greatly improving the measurement efficiency.

In the application of low-field unilateral magnetic resonance, due to its poor magnetic field uniformity and low signal-to-noise ratio, the parameters of T2 generally do not need to be selected, and the echo time needs to be set as small as possible. Therefore, this study does not involve the adaptive adjustment of the measurement parameters in the T_2_ experiment^30^.

## Conclusion

In this paper, the diffusion and relaxation curves of the samples are estimated by Monte Carlo simulation, and the algorithm selects the optimal measurement parameters according to the estimated values to achieve ${\mathrm{D}}_{0}$ and ${\mathrm{T}}_{1}$. The algorithm selects the optimal measurement parameters based on the estimated values and automates the parameter setting during the measurement, saves the measurement time, significantly reduces the measurement threshold, and facilitates $\mathrm{NMR}$. The algorithm has been experimentally verified to be able to obtain more accurate results than the previous algorithm. The algorithm has been experimentally verified to obtain more accurate measurement results. Notably, the proposed intelligent search algorithm is based on the premise of single-component samples, and it needs to be extended for multi-component samples, which is also the future research direction of our team.

## Data Availability

The datasets generated and/or analysed during the current study are not publicly available due private reasons but are available from the corresponding author on reasonable request.
